# Cooperation in Health: Mapping Collaborative Networks on the Web

**DOI:** 10.1371/journal.pone.0071415

**Published:** 2013-08-20

**Authors:** Pamela Barreto Lang, Fábio Castro Gouveia, Jacqueline Leta

**Affiliations:** 1 Coordination of Social Communication, Oswaldo Cruz Foundation (Fiocruz), Rio de Janeiro, Brazil; 2 Museum of Life, Oswaldo Cruz Foundation (Fiocruz), Rio de Janeiro, Brazil; 3 Medical Biochemistry Institute, Federal University of Rio de Janeiro (UFRJ), Rio de Janeiro, Brazil; University of Michigan, United States of America

## Abstract

**Objective:**

To map and investigate the relationships established on the web between leading health-research institutions around the world.

**Methods:**

Sample selection was based on the World Health Organization (WHO) Collaborating Centres (CCs). Data on the 768 active CCs in 89 countries were retrieved from the WHO's database. The final sample consisted of 190 institutions devoted to health sciences in 42 countries. Data on each institution's website were retrieved using webometric techniques (interlinking), and an asymmetric matrix was generated for social network analysis.

**Findings:**

The results showed that American and European institutions, such as the Centers for Disease Control and Prevention (CDC), the National Institutes of Health (NIH) and the National Institute of Health and Medical Research (INSERM), are the most highly connected on the web and have a higher capacity to attract hyperlinks. The Karolinska Institute (KI-SE) in Sweden is well placed as an articulation point between several integrants of the network and the component's core but lacks general recognition on the web by hyperlinks. Regarding the north-south divide, Mexico and Brazil appear to be key southern players on the web. The results showed that the hyperlinks exchanged between northern and southern countries present an abysmal gap: 99.49% of the hyperlinks provided by the North are directed toward the North itself, in contrast to 0.51% that are directed toward the South. Regarding the South, its institutions are more connected to its northern partners, with 98.46% of its hyperlinks directed toward the North, and mainly toward the United States, compared with 1.54% toward southern neighbors.

**Conclusion:**

It is advisable to strengthen integration policies on the web and to increase web networking through hyperlink exchange. In this way, the web could actually reflect international cooperation in health and help to legitimize and enhance the visibility of the many existing south-south collaboration networks.

## Introduction

Since the mid-1990s, the web has been widely explored to map and understand relations between organizations in different fields [1] and by various sectors of society [2]–[4]. Many studies have already recognized a positive correlation between networks of web pages linked by hyperlinks and networks of scientific collaboration linked by citations [5], [6]. Accordingly, several studies have shown a correlation between links exchanged between websites and relationships outside of the virtual world, especially within organizations or universities [7]–[10]. In fact, the web has been credited for its potential to provide new possibilities for enhancing and mapping cooperation [11].

In the era of globalization and when international cooperation in health plays an important role in reshaping global health, a better understanding of how health institutions are behaving on the web may greatly contribute to designing policies to help legitimize and enhance the visibility of existing collaborations on the web. Considering the emergence of webometrics, and given that the field of health has not yet been investigated with this approach, we designed an empirical study to investigate the relationship between health-research institutions on the web. The selection of these institutions was based on the World Health Organization (WHO) Collaborating Centres (CCs) and consisted of 190 institutions representing 42 countries. Created by WHO in 1949, these CCs have the purpose of integrating a collaborative network that conducts institutional activities to support programs developed by the WHO. As several of the CCs are among the world's most prestigious, leading health-research institutions, and as the CCs are present in a wide range of countries, we believe that mapping the relationships established by these institutions on the web may reveal the key players in this network, the least connected institutions and the possibility of a north-south division.

## Methods

The research was primarily based on webometric and social networking analyses. Webometrics is a research field devoted to understanding the construction and use of information on the Internet [12]. In this context, the hyperlink (a URL) serves as the main unit of analysis in webometric studies and is also considered to be an important indicator of the impact and relevance of web relations [8]. In this paper, interlinking data (that is, hyperlinks between institutional websites) were retrieved to map the number of exchanged hyperlinks between two or more websites. This type of analysis has proven to be very useful for studying institutional relations in the web environment [13]–[15].

With the purpose of better understanding and visualizing the links between institutions, the technique of social network analysis (SNA), derived from sociology, social psychology and anthropology [16], was applied to the data. The technique has been increasingly used in the field of information science [17] and is already being applied in webometric studies [18]. The nodes or actors present in the network can refer to individuals, organizations or groups connected by a certain type of relationship. These entities may play different roles depending on the position that each occupies in the network, such as cut-points, which are points of articulation between other elements that form the component [19].

There are two other basic elements composing the SNA technique: bonds, which may be weaker or stronger depending on the relative number of links exchanged between the nodes, and the flow of information, which corresponds to the directionality of the relationship, which is either uni- or bidirectional.

### Dataset

In the present study, institutions were selected based on the list of WHO CCs. Currently, there are approximately 900 CCs spread across 90 countries and six regions in which the WHO maintains offices: the Western Pacific: 21%, the Americas: 21%, Southeast Asia: 10%, the Eastern Mediterranean: 6%, Africa: 4%, and Europe: 37%. In the Americas, the United States contains the largest number of centers, with 99 centers, followed by Canada, with 25, and Brazil, with 21 [20]. CCs have a wide range of themes of research, which may vary from food contamination monitoring to health systems research and management. Considering the year of designation, the oldest center that is still active dates back to 1950.

On October 26^th^ 2009, data on the 768 active CCs in 89 countries were collected from the WHO's database, including the name of the CC, theme of the collaboration, contact person, institution, address, city, country, designation date, last designation and website.

As the data on all active CCs presented several inconsistencies, we established several methodological steps to define the studied sample. We started by excluding websites that did not match the name of the CC appointed by the WHO or websites that had changed or ceased to exist. Thus, the first step was to reduce the WHO's list to only those CCs that had a correct website address. Because CCs may include private institutions or research institutes, universities, departments or laboratories, the second step consisted of identifying the institutions to which these CCs belonged at a macro-institutional level. As a third step, as the selection of the sample considered the concept of websites as a set of pages within the same web domain, institutions whose websites were a subdirectory under a domain were excluded from the sample.

Lastly, institutions that were not exclusively dedicated to the field of health, such as universities, were also excluded, given the impossibility of analyzing the motivations that lead to a particular configuration of a network composed of institutions with very different research foci. It is noteworthy that because the current work is an institution-based study that used the list of WHO CCs as a criterion for sample selection, institutions with more than one center were represented in the sample by a single website corresponding to the main institutional domain. Hence, considering all of the previous criteria for inclusion/exclusion, the final list was composed of 354 institutions in 52 countries.

### Data retrieval and organization

Data on interlinks were retrieved between November 7^th^ and 9^th^, 2009. The numbers of interlinks between pairs of websites were obtained using Webometric Analyst [21] and the following string: “linkdomain:URL*i* site:URL*j*”. An asymmetric matrix was generated, and the diagonal was set to zero (Dataset S1).

At this stage, due to certain methodological requirements, we had to use a criterion of sample adhesion, so the sample was reduced once again. Considering the asymmetric matrix, institutions whose sum of the line number was lower than the total number of institutions within the sample (*n*) divided by two were excluded. In this case, the line number represents the number of hyperlinks received by each institution. Although the use of this criterion reduced the total number of studied institutions, the criterion provided a more balanced sample because all selected institutions presented a minimum level of required interconnection. This normalization process was performed successively until 190 institutions remained, based in 42 countries.

Data on the interlinks between the 190 websites were consolidated in a country-based asymmetric matrix. As there is no consensus or standard classification that considers the differences between developed, developing and underdeveloped countries, the aggregation into the North and South considered the economic development classification provided by the United Nations (UN) System [22]. Thus, countries classified by the UN as economically developed were grouped as “north”, whereas developing and underdeveloped countries and countries in transition were grouped as “south”.

### Network assembly, visualization and analysis

The asymmetric matrix of institutions was exported to UCINET, software commonly used in studies that apply social network analysis [23]. Networks were visualized with NetDraw, which is embedded in the UCINET package.

The following indicators were used in this study: Degree Centrality, which measures the number of lines incident to a node [24] and allowed detection of the most active institutions with a high hyperlink exchange degree in this study; Freeman's Betweenness Centrality, which measures the capacity of one node to help the connection of nodes that are not directly connected [25] and indicated the institutions with the largest capacity for attracting hyperlinks in this study; and K-Core, which detects different levels of centrality for each group [26]. Networks were visualized with NetDraw, which is embedded in the UCINET package. In this case, the diagonal was set to zero.

## Results and Discussion

**Figure 1:**
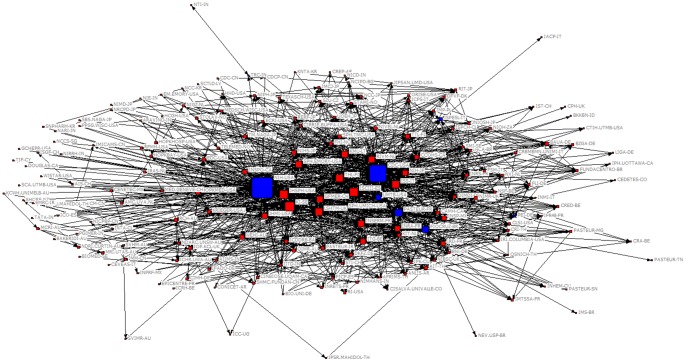
A web environment network for 190 health-research institutions in the world. The cut-point nodes are labeled as blue squares. The node sizes are set by degree.

Network analysis using UCINET presents the 190 institutions grouped into a single component, with a density measure of 0.59, meaning that approximately 59% of all possible ties are present (Figure 1). In the following sections, the main analyses are presented.

### Core and peripheral institutions

The component's core, according to K-Core measures, is composed of 23 institutions, which are considered to be key players in the network (Table 1). With the exception of two Latin American institutions (the Oswaldo Cruz Foundation – FIOCRUZ-BR; and the National Institute of Public Health – INSP-MX), the core is mainly composed of North American and European institutions.

**Table 1:** K-Core institutions by name, country and abbreviation.

K-Core Institutions	Country	Abbrev.
Bloomberg School of Public Health	United States	JHSPH-USA
Carlos III Health Institute	Spain	ISCIII-ES
Centers for Disease Control and Prevention	United States	CDC-USA
Geneva Foundation for Medical Education and Research	Switzerland	GFMER-CH
Institute of Tropical Medicine	Belgium	ITG-BE
Johns Hopkins Medical Institutions	United States	JHMI-USA
Karolinska Institute	Sweden	KI-SE
Kaunas University of Medicine	Lithuania	KMU-LT
London School of Hygiene and Tropical Medicine	United Kingdom	LSHTM-UK
National Institute for Agricultural Research	France	INRA-FR
National Institute of Health and Medical Research	France	INSERM-FR
National Institute for Health and Welfare	Finland	THL-FI
National Institute for Public Health and the Environment	Netherlands	RIVM-NL
National Institutes of Health	Italy	ISS-IT
National Institute of Health Sciences	Japan	NIHS-JP
National Institute of Infectious Diseases	Japan	NIH-JP
National Institute of Public Health	Mexico	INSP-MX
National Institutes of Health	United States	NIH-USA
Oswaldo Cruz Foundation	Brazil	FIOCRUZ-BR
Pasteur Institute	France	PASTEUR-FR
Rollins School of Public Health	United States	SPH.EMORY-USA
School of Public Health, University of Michigan	United States	SPH.UMICH-USA
University of Texas Medical Branch	United States	UMTB-USA

Among the peripheral institutions with lower K-Core values, the role of the Centre for Public Health (CPH-UK), the Person-Centered Approach Institute (IACP-IT), the Social Medicine Institute (IMS-BR), the Center for the Study of Violence (NEV.USP-BR), the National Tuberculosis Institute (NTI-IN), the Pasteur Institute of Tunisia (PASTEUR-TN) and the Thalassaemia International Federation (TIF-CY) must be highlighted. These institutions are only connected to the component by eight single institutions, which are acting as cut-points, meaning that these institutions are points of articulation to other integrants. If these connections did not exist, the institutions would be completely isolated.

Looking closely at several of these peripheral institutions, we found that the institutions' limited web connection does not reflect the scope of their international collaborations outside of the web. The TIF-CY, for instance, has established official relations with the WHO's Noncommunicable Diseases/Human Genetics Department since 1996 and represents 108 national thalassemia associations and other members from over 55 countries around the world [27]. However, the TIF-CY is included in the link network because of its single connection with the National Institutes of Health (NIH-USA), which is its cut-point. Other examples are the Centre for Public Health at Liverpool John Moores University (CPH-UK), recognized as the first institution to be an official partner of Global Violence Prevention [28]; the NTI-IN, which forms the Indian Health InterNetwork (HIN) for Tuberculosis and has been conducting several studies on tuberculosis [29], [30]; and the IACP-IT, Italy's focal point for the International Labour Organization (ILO) on Safety and Health at Work and the Environment [31]. As for the first example, these centers appear in the network because the centers are connected to a single institution: the CPH-UK is connected to the CDC-USA; the NTI-IN to the National Institute for Tuberculosis Research, formerly the Tuberculosis Research Centre (TRC-IN) in India; and the IACP-IT to the National Institute for Occupational Safety and Prevention (ISPESL-IT).

### Centrality measures (InDegree and OutDegree)

An analysis of degree measures indicated that institutions with the higher numbers of inlinks are not the largest providers, establishing an unbalanced level of institutional recognition. Both the InDegree (the number of links that lead into the node) and OutDegree (the number of links that lead out of the node) values for the top 20 institutions are shown in Table 2.

**Table 2:** The 20 top health-research institutions by their centrality degree measure (OutDegree and InDegree).

Institution	OutDegree	Institution	InDegree
National Institute for Agricultural Research (INRA-FR)	28,697	National Institutes of Health (NIH-USA)	113,449
National Institutes of Health (NIH-USA)	27,103	Johns Hopkins Medicine (HOPKMED-USA)	26,666
Centers for Disease Control and Prevention (CDC-USA)	17,317	Centers for Disease Control and Prevention (CDC-USA)	20,608
Johns Hopkins Medical Institutions (JHMI-USA)	16,777	National Institute of Health and Medical Research (INSERM-FR)	14,118
National Institute of Infectious Diseases (NIH-JP)	15,533	Bloomberg School of Public Health (JHSPH-USA)	4,079
London School of Hygiene and Tropical Medicine (LSHTM-UK)	14,825	Carlos III Health Institute (ISCIII-ES)	3,775
Geneva Foundation for Medical Education and Research (GFMER-CH)	10,843	Johns Hopkins Medical Institutions (JHMI-USA)	3,454
Center for Intern. Rehab. Res. Inform. and Exchange (CIRRIE.BUFFA- USA)	9,300	University of Texas Medical Branch (UMTB-USA)	1,436
Karolinska Institute (KI-SE)	7,537	National Institute for Agricultural Research (INRA-FR)	1,251
Howard County General Hospital (HCGH-USA)	5,290	Education Development Center (EDC-USA)	846
National Institute of Health and Medical Research (INSERM-FR)	4,750	Johns Hopkins Medicine (HOPKHOSP-USA)	751
Johns Hopkins Medicine (HOPKMED-USA)	4,121	Pasteur Institute (PASTEUR-FR)	676
University of Texas Medical Branch (UMTB-USA)	4,098	Research Institute for Development (IRD-FR)	534
Bloomberg School of Public Health (JHSPH-USA)	4,086	Oswaldo Cruz Foundation (FIOCRUZ-BR)	495
Institute of Psychiatry, King's College (IOP.KCL-UK)	3,102	National Institute for Public Health and the Environment (RIVM-NL)	472
National Institute of Public Health (INSP-MX)	3,033	Health and Human Development Programs (HHD-USA)	358
National Institute of Health (ISS-IT)	1,612	National Institute for Health and Welfare (THL-FI)	355
National Cancer Centre (NCC-JP)	1,475	Finnish Institute of Occupational Health (TTL-FI)	352
Pasteur Institute (PASTEUR-FR)	1,116	National Institute of Health (ISS-IT)	350
Oswaldo Cruz Foundation (FIOCRUZ-BR)	1,055	Karolinska Institute (KI-SE)	339

Only 11 institutions (the NIH-USA, HOPKMED-USA, CDC-USA, INSERM-FR, JHMI-USA, UMTB-USA, INRA-FR, PASTEUR-FR, FIOCRUZ-BR, ISS-IT and KI-SE) are on both lists, meaning that these institutions have a very well-balanced interconnection on the web by recognizing and being recognized by its pairs. The NIH is the institution with the highest number of inlinks but is second as a link provider. The CDC-USA maintains the third position on both lists. Nine institutions (the NIH-JP, LSHTM-UK, GFMER-CH, CIRRIE.BUFFA-USA, HCGH-USA, JHSPH-USA, IOP.KCL-UK, INSP-MX and NCC-JP), including one Mexican and two Japanese institutions, provide significant recognition to their collaborators but are not equally recognized, generating a gap in how this cooperation is reflected on the web. FIOCRUZ-BR is the only Latin American institution to appear on the top-20 list for InDegree, being ranked 14^th^.

An unexpected result came from the KI-SE, ranked 20^th^ in receiving hyperlinks from partners. The Karolinska Institute is the most central institution in the Swedish biomedical research system and the third most active European institution regarding the number of partners and collaborative projects and the percentage of funding received from the 6^th^ Framework Programme of the EU in the ‘‘Life sciences, genomics and biotechnology for health’’ thematic area [32]. In fact, despite its international recognition, the Karolinska Institute actually provides more hyperlinks than the organization receives, being ranked 9^th^ for the OutDegree measure.

The most active European institution according to the same parameters [32] is the INSERM-FR, with 956 partners and 164 ongoing projects solely in the EU. On the web, the INSERM-FR's international recognition can also be observed, even when considering countries outside of the EU. However, the French institution falls by seven positions (from 4^th^ to 11^th^ place) when being a link provider is used as the criterion.

### Freeman's Betweenness Centrality

Although not one of the most highly connected institutions on the web, according to Freeman's Betweenness Centrality measures, the KI-SE appears to be third, with a higher capacity for attracting partners and acting as a hub for connecting different institutions to the network's core (Table 3). The results point to the NIH-USA and CDC-USA as the institutions with the highest values for this measure.

**Table 3:** The 20 top health-research institutions by their centrality betweenness.

Country	Institutions	nBetweenness
United States	National Institutes of Health (NIH-USA)	46.229
United States	Centers for Disease Control and Prevention (CDC-USA)	21.595
Sweden	Karolinska Institute (KI-SE)	5.431
France	Pasteur Institute (PASTEUR-FR)	4.911
Italy	National Institute of Health (ISS-IT)	4.060
Brazil	Oswaldo Cruz Foundation (FIOCRUZ-BR)	3.366
Finland	National Institute for Health and Welfare (THL-FI)	3.317
United States	Bloomberg School of Public Health (JHSPH-USA)	3.178
Switzerland	Geneva Foundation for Medical Education and Research (GFMER-CH)	2.901
Spain	Carlos III Health Institute (ISCIII-ES)	2.498
Mexico	National Institute of Public Health (INSP-MX)	2.219
United States	Rollins School of Public Health (SPH.EMORY-USA)	2.159
Belgium	Institute of Tropical Medicine (ITG-BE)	1.869
France	National Institute of Health and Medical Research (INSERM-FR)	1.785
United States	University of Texas Medical Branch (UMTB-USA)	1.712
Canada	Public Health National Institute of Quebec (INSPQ-CA)	1.698
Netherlands	National Institute for Public Health and the Environment (RIVM-NL)	1.670
Australia	Australian Institute for Health and Welfare (AIHW-AU)	1.667
Japan	National Institute of Infectious Diseases (NIH-JP)	1.534
United States	Johns Hopkins Medicine (HOPKMED-USA)	1.431

### North-south division

Regarding international collaboration, the socioeconomic division between economically developed, industrialized countries, collectively known as the North, and low- and middle-income countries, known as the South, is an important subject of debate in the health field and represents a challenge, particularly in global health. To overcome northern dependence, to ensure the transfer of technology and to develop local health infrastructure, south-south cooperation has been continuously stimulated over time.

The limited expression of southern institutions in all of the analyzed parameters led to the question of a possible division between the North and the South on the web, by means of hyperlinks. The consolidation of data into a country-based matrix allowed a closer look on this matter (data not shown). Though the north-south relation has been criticized over the years for creating unidirectional dependence, in which the process of high-end technology transfer does not generate the infrastructures needed for the development of the local health system and health policies, such relation dynamics are still common in many cooperation programs [33]. To face the challenges facing north-south cooperation, many efforts have been made over the years to foster cooperative activities between newly industrialized southern countries and others in the south in order to find solutions to common development challenges.

Aside from FIOCRUZ-BR and its connections, other south-south collaborations, including those collaborations involving such donor countries as India, South Africa, Malaysia, Korea and China, are still not reflected by hyperlinks on the web. Institutions from low-income or even emerging countries appear on peripheral nodes, as these institutions are weakly related to the component's core, which is mainly represented by high-income countries. In this study, the North provided 86,568 links, representing 94.67% of the total number of links, in contrast to the South, whose 4,875 links represented 5.33% of the total. By analyzing the links exchanged between northern and southern countries, the results present an abysmal gap: 99.49% of the links provided by the northern region were directed toward the North itself, in contrast to 0.51% directed toward the south. In the South, institutions are still recognizing more northern partners as key players by providing 98.46% of the links to the North, and mainly to the United States, in contrast to 1.54% of the links to southern neighbors.

Interestingly, Mexico and Brazil stand out as link providers to the North. Mexico donates 2,841 links, representing more than half of the total links provided by the South, to a single northern country, Spain. In contrast, Brazil has the most well-balanced distribution of links to the North, presenting web relations with nearly half of the countries in the sample. Notably, despite the low percentage, when considering the directionality of links, south-south cooperation is three times higher than north-south cooperation. Although the northern region is still considered to be a major reference in health research, it seems that south-south cooperation programs are beginning to reflect the web structure to a certain degree.

### General Remarks

The goal of the present study was to map the relationships of 190 institutions, CCs and other health institutions on the web. The results showed that American and European institutions, such as the CDC-USA, NIH-USA and INSERM-FR, are the most connected on the web and have a higher capacity to attract hyperlinks. In contrast, the KI-SE, despite its worldwide recognition in the health field, is well placed as an articulation point between several integrants of the network and the component's core but lacks general recognition on the web by means of hyperlinks. Regarding the north-south divide, Mexico and Brazil present themselves on the web as key southern players. A predominance of north-north and north-south web relations in which the South provides most of its hyperlinks to the North, recognizing northern countries as key players in health research, was also observed.

Webometric studies have been expanding over the years and have been considered to be a very useful tool in many disciplines that recognize the importance of the web as an extension of real life-based research. However, one must realize that such studies do not necessarily reflect reality. Hence, any attempt to compare virtual and real pictures must consider several of the limitations imposed by webometric analyses.

In our study, we observed a lack of south-south relations reflected on the web, despite many existing successful south-south cooperation programs. Such contrast may be a consequence of the webometric criterion used for including/excluding institutions, which is a methodological step frequently used in webometric studies. In the present study, however, this may not be a significant problem as our final sample presented 22 countries representing the northern region and 20 countries in the South, a quite balanced scenario. Another important aspect to consider is the web environment and its usage by southern countries. It is well known that this set of countries has a less developed computational system than northern countries. Such a technological deficit may favor the construction of smaller, simpler structured websites with fewer pages and therefore fewer hyperlinks.

A final consideration is that as in any other empirical investigation, the data and conclusions in the present study exclusively refer to the 190 analyzed institutions. As this selective sample of institutions does not represent all health-research institutions, generalizations must be avoided.

Despite the limitations stated above, we believe that the results presented in this study represent a valuable portrait of the web network formed by several of the top research institutions in the field of health, contributing to possible further analysis and a plan for the strategic repositioning of these institutions on the World Wide Web. We particularly note the need to strengthen integration policies in the web environment and to increase web networking through hyperlink exchange. In this way, the web can actually be used to investigate international cooperation and to help legitimize and enhance the visibility of the many existing collaboration networks.

## Supporting Information

Dataset S1 Asymmetric matrix with data on the interlinks between the 190 institutions studied.
(XLS)
